# Point of Care Diagnostics in Resource-Limited Settings: A Review of the Present and Future of PoC in Its Most Needed Environment

**DOI:** 10.3390/bios10100133

**Published:** 2020-09-24

**Authors:** Benjamin Heidt, Williane F. Siqueira, Kasper Eersels, Hanne Diliën, Bart van Grinsven, Ricardo T. Fujiwara, Thomas J. Cleij

**Affiliations:** 1 Sensor Engineering Department, Faculty of Science and Engineering, Maastricht University, P.O. Box 616, 6200 MD Maastricht, The Netherlands; benjamin.heidt@maastrichtuniversity.nl (B.H.); williane.f.siqueira@gmail.com (W.F.S.); hanne.dilien@maastrichtuniversity.nl (H.D.); bart.vangrinsven@maastrichtuniversity.nl (B.v.G.); thomas.cleij@maastrichtuniversity.nl (T.J.C.); 2 Laboratory of Immunology and Genomics of Parasites, Department of Parasitology, Institute of Biological Sciences, Universidade Federal de Minas Gerais, Belo Horizonte 07262-130, Brazil; rtfujiwara@gmail.com

**Keywords:** point of care diagnostics, low-income countries, resource-limited settings, bottlenecks to usage by patients

## Abstract

Point of care (PoC) diagnostics are at the focus of government initiatives, NGOs and fundamental research alike. In high-income countries, the hope is to streamline the diagnostic procedure, minimize costs and make healthcare processes more efficient and faster, which, in some cases, can be more a matter of convenience than necessity. However, in resource-limited settings such as low-income countries, PoC-diagnostics might be the only viable route, when the next laboratory is hours away. Therefore, it is especially important to focus research into novel diagnostics for these countries in order to alleviate suffering due to infectious disease. In this review, the current research describing the use of PoC diagnostics in resource-limited settings and the potential bottlenecks along the value chain that prevent their widespread application is summarized. To this end, we will look at literature that investigates different parts of the value chain, such as fundamental research and market economics, as well as actual use at healthcare providers. We aim to create an integrated picture of potential PoC barriers, from the first start of research at universities to patient treatment in the field. Results from the literature will be discussed with the aim to bring all important steps and aspects together in order to illustrate how effectively PoC is being used in low-income countries. In addition, we discuss what is needed to improve the situation further, in order to use this technology to its fullest advantage and avoid “leaks in the pipeline”, when a promising device fails to take the next step of the valorization pathway and is abandoned.

## 1. Introduction

Low- and middle-income countries (LIC/MICs) face severe challenges due to limited economic opportunities. In addition to the economic struggles, LICs also bear a large burden of transmittable diseases, posing severe risks to the population’s wellbeing [1]. Healthcare systems and healthcare providers in LICs are often ill-equipped to treat the patients in the best possible way, especially in rural areas. Given the economical and infrastructural challenges in LIC, PoC diagnostics, which are often characterized by being independent of laboratory or medical infrastructure, as well as being highly affordable and holding considerable promise to improve the situation. Yet the actual commercialization of PoC diagnostic-tests lags well behind the innovative research and developments done in laboratories. 

**Figure 1:**
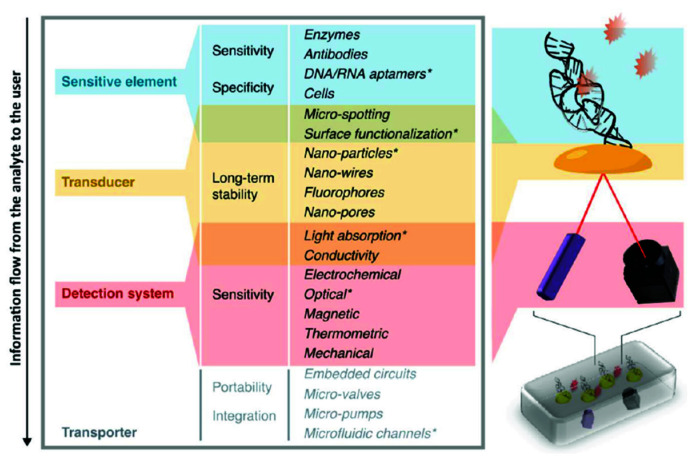
Migliozzi and Guibentif et al., looked at PoC bottlenecks from a technological standpoint. Figure re-used from [4] with permission under open-access creative commons copyright agreement.

**Figure 2:**
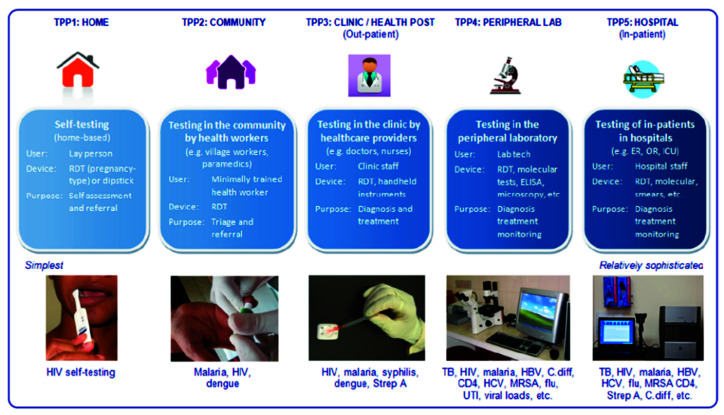
Pai et al., investigated PoC use within different usage scenarios. Figure adapted from [5] with permission under open-access copyright agreement.

Due to this strange dichotomy between promising, innovative research and very limited valorization into real products, several review articles on the topic have been written in past years. However, most are specialized on one specific aspect—for example some authors looked in depth at logistical shortcomings [2]; others investigated funding and collaboration considerations [3]. Reviews also summarized the topic in consideration of different viewing angles, such as the technological aspects and their implications, as seen in Figure 1 [4]. Another approach is to distinguish between different usage profiles, from the use at home up to the use in a laboratory, and argued that what point-of-care means depends tremendously on how and where it is used, which is shown in Figure 2 [5]. 

**Figure 3:**
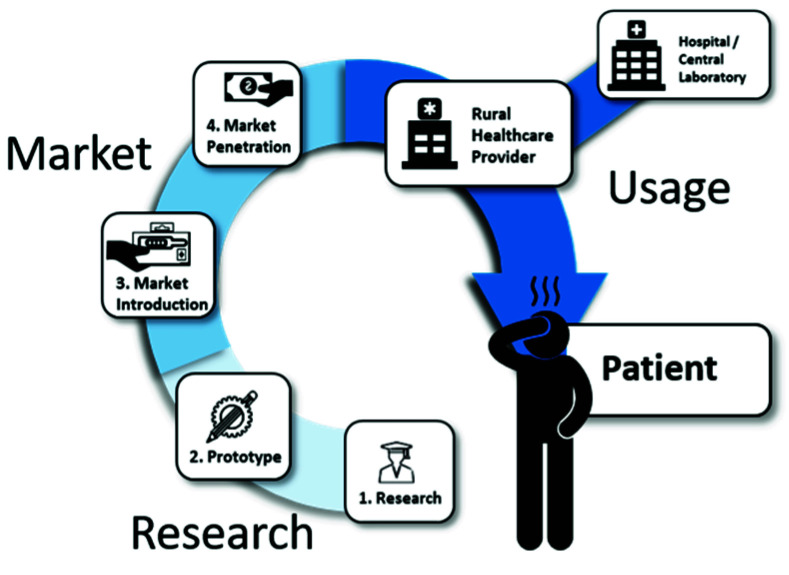
Segmentation of stages a PoC device has to pass to be able to bring a benefit to the patient.

This review aims to investigate the topic from a different angle. Here, we will look at the barriers of PoC diagnostics along the entire value chain, from the first idea in a laboratory to the use of the final product by a healthcare provider. With this review we aim to locate the “leaks in the pipeline” of PoC commercialization, that typically occur when PoC devices do not manage to proceed to a next step in the value chain. Such leaks include problems with funding that prevent the design of a prototype, or intellectual property (IP) considerations preventing market access. In this way, it may be possible to determine at which discrete steps the transition fails and a PoC device that was maybe once-promising becomes abandoned or underused. For this, the value chain of PoC devices is separated into three distinct domains, which themselves consist of separate steps. These domains are labeled research, market and usage, respectively, and any device has to achieve success in each of the steps within these domains to be able to reach the market and exert a benefit to the patient at the end of the value chain. These different domains in the value chain can be further subdivided into subdomains (Figure 3) with research—e.g., consisting of “Fundamental Research” or “Proof of Concepts and Prototyping”. In the second part of our review “The Market” is investigated, which looks at fundamental economic problems in the steps “Market Introduction” and “Market Penetration”. Finally, the “Usage Environment” will give an insight into the last barriers for actual use by healthcare providers. In this way, we aim to incorporate the entire value chain by examining both original research and review articles. 

## 2. Research

### 2.1. Fundamental Research: Funding Availability and Focus 

Fundamental research is the first step in the development of a PoC device and concerns all the basic research that is not yet directly related to a prototype or a product. Historically, the research of PoC devices started with simple dipstick tests with immobilized reagents—for example, for the detection of glucose [6]. Later the laboratory use of immunoassays, especially the high sensitivity of radioimmunoassays and enzyme-linked immunoassays, created interest in improving those methods into rapid tests, which eventually became lateral flow immunoassay, the most abundant type of PoC device [7]. While lateral flow devices are the most common, the trend in research goes towards devices with higher complexity, which are able to handle more complicated samples, can multiplex and detect challenging analytes [6]. The greater complexity of these devices is shown in their mechanisms of action, which are often optical, electro(chemical) or magnetic [8], but also include other mode of actions—e.g., thermal sensor systems [9,10]. Optical systems include more straightforward UV/Vis or fluorescence sensors, as well as more complex systems using quantum dot and surface-plasmon-resonance technology or even genetically encoded biosensors [11,12,13,14]. Examples of electro-(chemical) systems range from basic ampere or voltametric systems over graphene-based field-effect-transistors to DNA-annealing-based redox-reporter assays [15,16,17,18]. However, compared to the abundance of new research, the actual commercialization lags behind and market examples welcoming these new innovations are rare [19]. 

#### 2.1.1. Choosing a Suitable Design Philosophy

There are several factors that may misguide new developments, an important one being the design philosophy of new devices. With respect to this issue, a stark contrast from the research philosophy in high-income countries (HIC) and the needs of LICs is evident. Research in HICs is rarely aimed at inexpensive technologies with wider impact [4,20], which are direly needed in resource-limited settings. Instead, research in HICs tends to focus on high-efficiency devices with even higher complexity, which is not a problem for affluent countries with good infrastructure; however, due to their complexity, such devices are often not usable (or affordable) in LICs [21]. Several studies point out the differences in approach between high-complexity PoC diagnostic test platforms (HCTs), such as the GeneXpert and low-complexity PoC tests (LCTs), such as widely available lateral flow tests–for example, for pregnancy and malaria [4,20,21]. While HCTs can conduct more difficult and sensitive diagnostic tests, they are also more complex and thus require more training, maintenance and infrastructure. LCTs, on the other hand, lack sensitivity and diagnostic power, but are highly affordable and low maintenance. 

This is not the only example of different design philosophies and approaches. In the design of biosensors in HIC, the most suitable biomarker for a given illness is chosen without much regard for the needed infrastructure. The ability to perform a venipuncture, for example, is a given in HICs, which means tests developed have no strong restrictions for the needed sample size. This can pose a problem for LICs in rural settings without a trained phlebotomist as venipuncture is not possible and the test has to be able to work either with easier to acquire samples, such as sputum or urine, or the much smaller blood sample quantities of a finger prick or heel-stick samples. Heel-stick sample sizes are usually under 5% of the size of venipuncture samples [22]. This illustrates that the research approach for LICs needs to be different. Instead of building a system around the best biomarker, the system needs to be created around the available infrastructure first, which is a considerably different design philosophy [22,23]. 

#### 2.1.2. Taking Aim: Proper Target Analytes

Next to the different design philosophies, the target of research is different in HICs and LICs. Resource-rich settings do not focus on research on neglected tropical diseases (NTDs), which is already evident from their name. The burden of NTDs is mostly nonexistent in affluent countries; instead, they predominantly affect the world’s poor, and are therefore less interesting for commercial research, as the chance of return of investment is limited. There is little incentive for affluent countries to deal with many NTDs, mainly due to the low impact these diseases have on the population of these countries [24]. This is also visible in drug development where, from 1975 to 2004, only 1% of drugs were developed for NTDs (21 out of 1556) [3]. 

Other gaps in knowledge might be easier to miss—for example, local differences in diseases, such as geographical variability in antigen presentation and DNA/RNA signals. In addition, other diseases prevalent in LICs can have an effect on a target analyte. For example, immunosuppression due to HIV leads to lower host response signals and reduces the sensitivity of some nucleic acid tests for the detection of pulmonary TB [25,26]. Another report remarks that, even when there is willingness, funding and know-how about adequate research and design philosophy, there are practical problems as simple as not being able to acquire appropriate samples for assay development [26]. In general, research can only make an impact in LICs if a bottom-up approach is used that takes the infrastructure and environment of LICs into account from the start [4].

#### 2.1.3. Funding in LICs

Research in LICs themselves is beneficial as it focuses directly on the regional circumstances and problems, with a LICs-centered philosophy. However, PoC funding in LICs themselves is highly inconsistent [20,27]; this leads to dependence on other funding opportunities, such as NGOs and development partnerships. This is not only the case for funding of PoC-research, but also for treatment-research and treatment itself. For some countries in Sub-Saharan Africa, HIV expenditures are strongly reliant on external sources, despite this being one of the region’s most important health risks. Kenya and Uganda contribute less than 15% of funding to their own national HIV-relief efforts, Mozambique even contributes only 3% [20,27]. In cases where there is secure funding through outside sources, it naturally tends to focus on treatment or prevention—e.g., vaccine development—while diagnostic research receives much less funding, leading to an over-reliance on clinical symptoms due to a lack of adequate diagnostics [4,28]. 

#### 2.1.4. Incentives to Change Focus in HICs

Possible solutions are new programs that are being implemented to incentivize product development for neglected tropical diseases [3]. However, such programs are mostly aimed at drug development and include, amongst others, the “Priority Review Voucher (PRV)” program in the United States, which gives out transferable priority FDA-review vouchers. It is one example of an implemented “pull-mechanism” to motivate development in neglected areas [3,29,30]. Next to such pull mechanisms, "push-incentives", such as the Global Health Investment Fund (GHIT), a partnership that connects the Japanese Government, several NGOs, as well as large drug and diagnostic manufacturers, and targets poverty-connected diseases and NTDs [3,31]. Other specifically named initiatives are the “Global Health Investment Fund (GHIF)” and the “Wellcome Trust Pathfinder Award” [3]. Incentive programs such as this may have a large effect when used specifically to incentivize PoC diagnostics. 

NGOs—e.g., the Bill & Melinda Gates Foundation—have contributed a substantial amount of funding for NTDs research and play an important part in integrating into such programs [32,33,34,35,36]. Having large industrial PoC developers on board may not only improve the device situation but may have further benefits in implementing the devices from a market penetration standpoint (vide infra). An increased cooperation due to the mentioned pull mechanisms can also help to prevent a “HIC-Bubble” when stakeholders from LICs are involved. 

To change the philosophy of research, “Frugal Development” must be considered from the start to build devices with relatively low complexity that are not only affordable and usable in LICs settings but also maintainable and repairable [37,38,39,40]. This not only serves as a base requirement, but also makes several aspects, such as acquiring spare parts and stock management, more straightforward down the line. Additionally, 3D-printing and other new technologies might be of use to create spare parts on demand, as those new devices have already been shown to be able to create simple microfluidic parts, and were able to supply hospitals with respirator valves during the COVID-19 pandemic [41,42].

### 2.2. Proof-of-Concept and Prototypes: The Importance of Appropriate Device Characteristics.

#### 2.2.1. Device Characteristics

Engineers in affluent countries tend to design devices that assume HICs infrastructure standards, meaning well-funded laboratories in regulated environments with quality control. This can be problematic in LIC, and therefore special design considerations must be taken into account [20]. Devices that perform well in controlled settings, in which the prototype is tested, often fail when challenged with tropical conditions in LICs [26]. Especially in rural settings, where access to, for example, electricity, might be problematic in terms of powered PoC devices [22,26,43], but also a lack of cold storage options, can form a significant challenge [9,22,23,43]. Limited refrigeration and power supply therefore demands that the device and its disposables have to be stable in the long term, even at high temperatures, while powered PoC devices need to be able to run on battery or solar [4,26,44]. 

#### 2.2.2. Being ASSURED

Many authors, agree that the device characteristics that are beneficial are following the WHO guidelines for PoC diagnostics, which are symbolized by the acronym, ASSURED; which stands for Affordable, Sensitive, Specific, User-friendly, Rapid and robust, Equipment-free and Delivered to those who need them. These requirements are regularly mentioned in publications [20,22,45,46,47,48], but it also has to be noted that these are ideal and strong requirements that only a selected few devices can meet [21]. 

While progress has been made on PoC tests for syphilis, chlamydia as well as gonococcal infections, there is still not one test available that complies with all ASSURED criteria [46]. Especially good sensitivity and selectivity, which are two of the most important points, get increasingly more difficult to achieve the closer the system is to a “perfect” system with an accuracy of 100% [21,22,26]. In rural LICs settings, a PoC diagnostic tool needs to be small, portable and highly affordable [43]. While many authors put great emphasis on affordability, others take a different position and argue that Zero-Cost is not an important parameter; arguing that reliability and standardization are seen as more crucial, and remark that it might be misguided to assume that “poverty reduces the value individuals place on their well-being” and therefore focus should be put on efficiency and reliability before anything else [28].

#### 2.2.3. How Necessary Is It to Be ASSURED? 

Many scientists cite ASSURED as a necessity and a large consensus for the ASSURED criteria among healthcare workers in, for instance, Uganda, has been found [49], Pai et al., give another, more pragmatic and contextual viewpoint. ASSURED, they argue, imposes artificial restrictions that may not be necessary, depending on the context in which the devices are used. For example, a device that is used for first line screening with the aim of referral to another, more specialized, healthcare provider for further diagnosis and treatment, can have a lower specificity compared to a test that makes decisions about, or monitors, the treatment itself [5]. Therefore, seeing efficiency and reliability above all might be shortsighted. This is also indicated by other research; in a study that compared 12 different combinations of hepatitis C virus-diagnostics that were either PoC, lab-based or a combination. The cheapest strategy turned out to be a two-test combination system, first using a lower-specificity PoC antibody test followed by a confirmation via an RNA PoC test. All one-step strategies showed higher false-positive rates and were not cost-effective under base-case assumptions. However, two-step strategies are highly dependent on the loss-to-follow-up rate (LTFU) [50]. Gift et al., reported as early as 1999 on the “rapid test paradox” and the connection between LTFU and PoC tests with limited sensitivity that lead to better treatment outcomes [51]. Other authors also agree with this notion, and argue that a very affordable test and one with very good stability at high temperatures, but with suboptimal sensitivity, may still be extremely beneficial in tropical settings [26].

Another aspect, indicating that focus on raw diagnostic prowess might be overrated and the important part may be the accessibility to diagnostics in the first place, is found in the fact that only 28% of the inhabitants of Africa have access to advanced healthcare facilities. Tests that need only minimal infrastructure could give an additional 47% of the African population access to diagnostic tests. While improving the accuracy for bacterial pneumonia tests in advanced healthcare facilities only led to 119,000 more disability-adjusted life-years (DALYs) saved per year, 263,000 more DALYs could be saved annually if this test would be made available for rural sites with minimal resources [43]. This was also found in another study, using syphilis as an example. A PoC device, that would require minimal laboratory infrastructure, could prevent 138,000 congenital syphilis cases and 148,000 stillbirth per year. A PoC device that would not require any laboratory infrastructure at all would prevent 201,000 cases and 215,000 stillbirths [22]. In the end, a perfectly assured device might be unrealistic, as several of the ASSURED criteria work against each other and especially against the affordability. The higher the accuracy the less affordable the device will be. Therefore, it is important to choose the right battles. 

Another aspect criticized is that PoC tests often focus on a single disease; however, healthcare workers in LICs are concerned with syndromes of unknown etiology [20]. Additionally, having one test measure several factors would give the healthcare provider more diagnostic security, and save time. 

#### 2.2.4. Steps towards an Effectively Usable PoC Device

There are several possibilities that can help tackle those challenges. Multiplexing might be a large step forward, and nowadays several methods for multiplexing are possible and being developed [4,22]. This would reduce the relative costs for each test since they can use the same framework and are regulated at the same time. Healthcare workers are often interested in many different factors connected to one disease. Putting them together in one test can show hugely beneficial they can be. Multiplexing also has logistical benefits, as there is less to track; associated equipment can be shared and several tests can be made while worktime is not increased.

Several authors suggest modern microfluidics to alleviate some of these problems. In sample preparation, for example, all the technology of DNA/RNA extraction and purification can be included in a small cartridge with very easy usability [43], reducing the need for external equipment and thus reducing cost [21]. The improvements in biosensor and microfluidic creation already helped make HTP devices possible due to the optimized microstructuring of transducers (nano-wires, nano-pores, nano-particles), microspotting of the sensitive element and integration in low volume microfluidic channels [4]. Many other authors agree and see great potential in microfluidics for LIC-PoC [52,53,54,55]. The widespread use of smartphones is considered in many publications as a possible readout-device for optical platforms, as well as an ICT-connector [56,57,58,59,60].

To prepare nucleic assays for LICs environments, one major problem is assay stabilization, as PCR mixes require cold storage. Lyophillization would be an option to create assays that do not require a cold-chain. The problematic part is the diverse array of compounds used that are incompatible with freeze-drying, such as glycerol. There are mixes that are possible to freeze-dry; however, the amplification efficiency will likely suffer [43]. To improve ruggedness, functions should be reduced to the essential and have integrated quality control, and local production may help with access to support and consumables [61].

## 3. The Market 

### 3.1. Market Introduction 

The step from prototype towards market introduction seems to be the most taxing step, as even low-cost PoC devices are being developed in many laboratories in HICs, but not being adopted as well in LICs [28]. 

#### 3.1.1. Funding and IP

Funding is not only a consideration of research, but also of valorization. Even when funding for novel research is available, additional funding for economic aspects, such as manufacturing, distribution and maintenance are harder to find, resulting in the abandonment of projects by the original researcher due to cost, as well as deterring investment and interest from companies, which favor secure, established products [26]. Bringing new medical devices to the market is a costly enterprise. This is investigated more in depth in drug development where it is estimated that developing a new drug exceeds USD 1 billion in cost, takes more than 10 years and only 11.8–21% of new drugs are approved. There are no discrete numbers on this for medical devices, given that the field is highly diverse with an estimate of over 500,000 different types of devices, which span from X-Ray machines to hip implants with different diverse aspects that have to be taken into account for regulation, such as repair, maintenance and wear [62]. These processes can take years until a patient reaps the benefit of a new product, if it reaches the market after all [63]. This might make it unprofitable for a company to conduct fundamental research for LICs PoC if there is not an immediate benefit in reach [3].

Next to funding, intellectual property (IP) proves to be a large barrier for the development of systems upon existing technologies. Especially IP on molecules and genes make this increasingly difficult [4]. Existing patents covering biomarkers and even entire organisms exist, and there are large IP barriers in diagnostic platforms and/or components of diagnostic platforms [21,22]. One solution for IP considerations are new private–public partnerships, such as the World Intellectual Property Organization (WIPO) Re:search consortium. WIPO is the UN agency tasked with developing international IP systems fostering innovation for NTDs that benefits everyone. WIPO Re:search enables members royalty free use of infrastructure, compound libraries, IP assets and know-how. The WIPO consists of 107 members in 30 countries. IP Licenses can be used royalty free and new IPs generated are retained by recipient member, and not the member of the original IP asset. Often, WIPO Re:search members are from LICs. This creates benefits for everyone, and LICs contribute local know-how and have access to patient samples, while companies, often from HICs, contribute their research power and IP. The company benefits from heightened corporate social responsibility, access to other IP, business opportunities and access to other experts and know-how in the field; as well as networking possibilities for LICs, which are emerging markets [3,64,65].

#### 3.1.2. Regulations

High regulatory barriers and strict healthcare standards are also a significant barrier to the introduction of innovations [4,5,20]. While good regulations are important, they are difficult and require substantial expertise to navigate them, which discourages innovation, especially in LICs where profitable returns are unsure. Clear and straightforward national policies for diagnostic evaluation and certification are key [20]. The pharmaceutical world is more advanced in setting up harmonization infrastructure, which is still lagging behind for diagnostics. In total, 23 countries in Africa banded together and pledged to harmonize the approval method for diagnostics, which is a huge step, as companies do not have to acquire approval for each country separately [66]. One other possible solution offers a WHO program that examines the quality and the safety of HIV and Malaria tests. This “prequalification” is a helpful guide for LICs, helping governments to speed up the approval process [26].

#### 3.1.3. Integrated Market Expertise

Missing collaboration between academia and industry might be another bottleneck encountered when bringing inventions from the lab to the market. A multidisciplinary team for market introduction is, just as it is for research, an important part of the valorization trajectory [4,21]. A study of 358 medical devices for LICs found that only 134 met the study requirements to count as “commercialized“ [61]. In addition, of the hundreds of devices beyond this particular study, many likely failed to commercialize because of, among other reasons, a failed transition from prototype to market introduction [61]. Studies have also indicated that good policy plans for quality systems and supply chains improves the accessibility if placed prior to the POCT program [20]. Those aspects of quality control and assurance and supply chain managements are what companies have strong expertise in. Those aspects are usually not considered enough in the prototyping phase, while scaling up is still not around the corner, this becomes a barrier later on into commercialization (vide infra). The expertise of companies can help to navigate the complexities of scaling and implementing a medical device and effective delivery mechanisms [21,61].

For this, it is not only necessary to have a research and valorization team, but also specialists for regulation, culture and policy [21]. Especially researchers in low-income-countries often lack the knowledge spanning all involved fields, from discovery and research to market introduction [3]. The device must consider local and regional constrains, involved stakeholders and their needs and the capacity of the local healthcare workforce, but also social and cultural contexts for things that can easily be overlooked—e.g., whether blood sampling is easily accepted [5,22,61]. One target group that should be especially focused on, but are often not included, are the end-users, who should be integrated in each step of the design process [20,28,45]. To this end, limited international (especially transcontinental) collaboration is problematic [21].

### 3.2. Market Penetration

#### 3.2.1. Device Quality

After the introduction of the product, other aspects become important for wider market penetration. One problem is a bad batch-to-batch reproducibility especially in lateral flow devices that hamper upscaling [21]. Low device quality and low reproducibility can lead to decreased trust by healthcare providers and thus affect the adoption of other devices negatively [28]. This is one of the greatest concerns of rural LICs healthcare practitioners. In interviews, key-stakeholders expressed the need for diagnostic scale-up, but they also had concerns about reliability, as well as supply chain management and staff training [45]. This was confirmed by another study, reporting doubts from healthcare practitioners regarding test trustworthiness with either the accuracy, the robustness or the clarity of results. Adding to distrust is the concern of counterfeit tests being delivered [49]. Participants of the study found PoC critical for improving healthcare but judged their current form as not suitable for the local context. 

#### 3.2.2. Economic and Social Placement 

Even a theoretical PoC device that achieved all ASSURED criteria perfectly might not be sustainable and gain market acceptance if they do not have a viable business model attached to them [45]. It is important to consider that this model fits in with the real-life workflow patterns. This means the PoC devices need to be integrated into the existing healthcare settings and real-life contexts of LICs to be successful. 

The large importance of proper economic placement is shown by the fact that even inaccurate devices can be a market success if the stakeholders along the value chain profit from their use. For example, inaccurate serological tuberculosis tests, which the WHO advises against, are very common in private healthcare facilities in India, as well as in 17 of the top 22 other countries most affected by the disease. Private doctors earn referral money and other incentives for ordered tests, leading to an overreliance on inaccurate diagnostics due to economic incentives [5,67]. While quality is of course of the utmost importance, this example shows that, for market penetration, the importance of correct economic placement and integration into existing value systems is a key factor, which sadly can even outweigh actual clinical performance. Therefore, when aiming at maximum market penetration, the whole market with all economic influences, costs and workflow has to be understood. This is just as important as delivering a device with the best quality possible [45]. 

Other examples of misplacement of PoC testing in the market can be found in India and South Africa, where many well-working and cheap (USD 1 per test) PoC tests are available for different diseases, such as HIV, Malaria, Dengue, Syphilis and Hepatitis. Yet many of those tests are still not commonly used at home, the physician’s office or even in rural healthcare clinics—arguably the target environment for PoC. Instead, testing happens foremost in laboratories and hospitals, whereas small independent laboratories are the major users of PoC devices [5]. Lab personnel are often skeptical against testing outside of controlled settings, as they lose control over testing and quality assurance and it interferes with their business model [20,45]. This shows how devices that are meant to be PoC can, due to mismatching interests in the established market, end up in a position where they cannot fulfill their intended purpose. 

The placement of a new PoC device into an already existing healthcare system requires satisfying challenges and questions raised by every stakeholder. Who bares which part of the financial cost? What economic incentives are offered to various stakeholders? How is the training handled and how are information and communication technology used for reporting? Answering these questions in a satisfying manner, might be as crucial as the device itself [5,45,68]. Clear national guidelines for essential steps—i.e., evaluation, certification, supply chain management, financing, training and expertise—need to be put in place [20]. It also has to be pointed out that healthcare providers often feel that they do not have any influence on the decision regarding the availability of PoC devices, but instead “use what they are given” [49]. This shows that they are often not taken into account in this process. Healthcare practitioners expect decision makers to lay out the plan and define the use of PoC devices in the context of local epidemiology. Decision makers also need to take care of training before deployment and give adequate guidelines on how to proceed after a positive or negative results [49]. 

Market penetration is also heavily dependent on price. Here, the willingness to pay is an important aspect. A guideline to evaluate the affordability is 1–3× the GDP per capita per quality adjusted life years, which is gained through the intervention [50]. However, this calculation is not commonly shared as a good estimate and it may not lead to the best investment if it means loss of funding in other areas. Society might not be willing to contribute the necessary sums just because of cost effectiveness [69,70]. Another factor might be that patients themselves are not willing to pay the suggested price as they lack money and cannot invest in the long-term benefits. In Kenya, 51% of healthcare expenses are payed out of pocket, healthcare costs are often covered through “harambees“ fundraising events in the community; 46% of the population only has USD 1 or less to spend each day [28]. HCTs platforms have high fixed costs for the PoC device itself and, therefore, the cost per test is strongly dependent on the use case and the workload of the device to be economically justifiable [71]; therefore, those diagnostic devices, with their high implementation cost, are considered as too expensive for widespread use [70]. A good example for this is HIV diagnosis and monitoring. Since viral counts are difficult to do in the field, often a laboratory procedure is required. While PoC HCTs devices are established on the market, widespread use is limited by the initial high cost of the device. However, research showed that it can be viable for clinics that have a moderate or large amount of patients to have such PoC HCTs devices for HIV detection, as the initial high cost of the device can be distributed over more patients. This is also true for the usage time. The longer the device can be maintained, the more cost-effective it can be. With 50 patients per month, a reasonable assumption for a clinic in South Africa, the overall cost for anti-retroviral therapy (ART) monitoring would be only USD 45 higher (USD 210 compared to USD 166) than the laboratory procedure over a time span of 5 years. However, assuming only 10 patients per month, this would increase to a USD 183 additional cost over the same timeframe. Price is also dependent on which biomarker is analyzed, as viral load was, in the 50 patients per month case, just as cost effective as in the laboratory, while CD4+ count and creatinine test were more expensive [71]. The notion of cost effective PoC ART monitoring is supported by several mathematical simulations from South Africa, Zimbabwe and Mozambique, especially when all the costs are taken into account. A PoC test that enables better ART linkage can be tremendously more expensive per test, and still save follow-up costs in the long run, due to better immediate and consistent treatment, as well as greater reach [72,73,74].

While many simulations of cost effectiveness base their assumption on high-prevalence areas, others remark that especially for “the last mile“, in areas with lack of infrastructure, PoC might be one of the only viable alternatives for the hardest to reach 10% of patients, as transport networks get more and more difficult to establish in remote areas [75]. Despite the low volume of patients making cost-effectiveness more difficult, it is estimated that an optimal placement of PoC viral load tests on-site and in PoC hubs still can reduce the price of a test by 6–35% by avoiding high transport cost in remote areas [76]. Finally, it has to be noted that, although PoC tests could significantly improve the healthcare system in LICs, their impact will depend on the specific disease or condition they are employed for. Therefore, the successful implementation of PoC will require a rigorous study of the overall cost–benefit ratio of any proposed PoC test, specifically addressing the disease it is meant to diagnose. 

#### 3.2.3. Product Distribution 

Limited infrastructure in LICs not only results in low return-of-investments for companies, but also makes the distribution of the device and technical support more difficult, which might discourage companies or hamper the market penetration [26]. Stock outs and supply network problems are a massive obstacle to market penetration. Studies on PoC accessibility and supply chain management reported several stock outs of PoC devices. In a scale-up program for syphilis tests from an NGO-led pilot to a ministry of health operated large scale operation in Zambia, half of the pilot sites suffered at least one stock out. PoC for pregnant women also reported stock outs in several stages of the study, with up to 60% of sites reporting stock outs. The longest time the device was out of stock was a median of 6 weeks. In Uganda, malaria diagnostic tests were only available in 24% of 125 lower healthcare facilities, and 72% of community healthcare workers did not receive malaria testing kits for 6 month [2]. In antennal clinics in Guatemala, almost half of women could not be tested for HIV, Syphilis and Hepatitis B, in part because of stock outs [2,77]. Test kit stock outs are also reported from Uganda and Tanzania [78] and are a major concern to healthcare providers [49].

Here, the supply chain is a big point of failure, mostly due to irregular supply, poor forecasting, selection of diagnostics, insecure procurement systems, delayed distribution systems, poor quality assurance and inadequate stocks [20,79]. This has also been confirmed by healthcare workers who are concerned about the reliability of the supply chain [45]. Human resources are often not considered in the supply chain management, leading to bad planning and overstretched systems [66]. 

An innovative solution for the fast distribution of medical products in LICs is shown by the company Zipline, which uses remote drones to distribute blood preservations to hospitals in need all over Rwanda. Replacing the delivery from taking hours by Motorbike, to mere minutes via a Zipline drone, this innovation reduces the number of blood stocks the hospital needs and thus reduces waste due to expiry. In emergencies, matching blood can be delivered within minutes, something impossible with motorcycle rides that could take up to 5 hours [80,81]. In Zipline’s system, the blood packs are dropped from the drone via a little parachute, hence a pickup of blood-samples from remote areas is difficult, since the drone cannot independently land. However in the recent COVID-19 pandemic, Zipline collected COVID-19 test-samples by car and sent them bundled together to large hospitals via their drones, while also distributing to hospitals other COVID-19 related necessities [82,83]. Innovations such as this might help to counteract shortcomings in delivery-planning and infrastructure or deliver tests and consumables that have limited shelf life or limited temperature resistance. A pickup service for test samples in rural healthcare clinics, for further diagnostic procedures—for example in a two-step process—might be possible with new developments of drones that are capable of vertically taking off and landing, and are currently developed, for example by DHL [84].

## 4. The Usage

Limited testing capabilities are often a bottleneck for adequate therapy. This is especially observable in HIV treatment, where CD4+ counts and viral load are used to monitor antiretroviral therapy (ART). In Sub-Saharan Africa, the median of patients that were retained between HIV diagnosis and CD4+ count was 59% [85]. HIV is a good example of a disease that is difficult to get a conclusive diagnosis on in the field as it needs a nucleic acid test that usually has to be performed in a laboratory with trained personnel [86,87,88]. While there are several HCTs PoC platforms available that can conduct HIV monitoring, such as the GeneXpert (Cepheid), the PIMA CD4+ (Abbot) or the Alere q (Abbot) [89,90,91], their high initial cost are still a problem. This makes ART therapy challenging to start and monitor in rural areas. Therefore, not only is supplementation with tests an important factor, but also the whole infrastructure of usage in combination with treatment, especially in rural healthcare settings where the different parts need to act together to create sensible plans for PoC testing and treatment delivery. Due to this interconnectedness of different factors surrounding the end-users, several bottlenecks can appear. HIV is, therefore, a good example of how healthcare, as well as patient management, are integral factors and can negate any positive effects that PoC can bring, if they are mishandled.

### 4.1. Healthcare Management 

Political will towards PoC might be reduced when PoC tests lead to more demand for treatment, while at the same time treatment capabilities are scarce [5]. On the other hand, PoC diagnosis might not be feasible (for a test and treat scenario) when adequate treatment capabilities are not in place [70]. When treatment is available, patients might also just opt-out of tests in favor of direct use of medication, such as over-the-counter-antibiotics. This has been reported in Thailand, where missing information about disease origin among the public leads to a preference of medication instead of proper diagnosis, as medication is connected to symptoms instead of disease origin. For example, patients associate antibiotics with the symptoms of a bacterial infection instead of the infection itself and thus demand antibiotics even when the reason for the illness is not bacterial in nature but has similar symptoms [92]. PoC testing might offer proper diagnoses, preventing people from self-diagnoses and taking inadequate medicine. This also shows the importance of looking at PoC applications not independently but in the whole context of the healthcare system, that acts and is acted upon by various factors. Some researchers assess that the introduction of a PoC device into a system that is already in place changes the role from a technical to a social device. However, this also has its upsides—a more elusive benefit of why healthcare workers in rural areas might want to use PoC testing is the psychological effect it can have on patients, giving healthcare workers more certainty which, in turn, transfers to the patient and improves compliance. Patients also might overestimate the capabilities of the tests, which encourages compliance [92]. PoC can provide evidence without the need of laboratory infrastructure and highly trained lab technicians [21].

### 4.2. Patient Management 

In addition to the infrastructural bottlenecks related to the availability of electricity and water, which were discussed in the research section, one of the most immediate problems is patient management. In rural areas, traditional laboratory diagnostics are limited by distance to central laboratories. For laboratory diagnostics, samples, for example, in the form of dried blood spots, have to be transported via motorcycle, creating problems of long turnaround times for test results of up to two weeks and the danger of sample damage or loss. Another problem is the loss-to-follow-up when patients have to either wait for their results or have to go to another facility. This may be a problem for two step diagnostic processes in which the second step is not PoC and the patient has to return or travel to a second facility [50]. Especially in rural LICs areas, there are large barriers to get to healthcare facilities, due to poor transport infrastructure and time constraints that might prevent a second visit [70]. 

For example, a third of the women in Ghana live further than two hours away from facilities with the capabilities of emergency obstetric and neonatal care [20,93]. Barriers such as these create gaps in the diagnostic and treatment pipeline and can lead to high levels of LTFU if patients do not return to collect their results and start treatment [44,88]. However, this also shows that highly effective laboratory diagnostics might not be suitable in LICs, even if their sensitivity and selectivity is far superior to PoC devices. If they pose the risk of LTFU, a one-step PoC device might be the more pragmatic and better solution [46]. In HIV diagnostic and treatment, the decentralization of diagnostics from large hospitals to rural healthcare centers (RHC) proved essential to give people outside of urban areas access to therapies, such as ART. Before the implementation of testing in RHCs, the rate of LTFU was unacceptably high [94] as over half of patients did not return to get their results [95]. 

The large potential of PoC diagnostics in this context is, in part, due to their ability to ensure the start of treatment in the same encounter, which is essential as the rapid initiation of treatment is immensely important in diseases such as aids and tuberculosis [45]. In India, just one round for combined screening and treatment of HPV reduced the cervical cancer rate and mortality for over 30-year-old women by 50% [96]. For this reason, the WHO recommends a screen and treat strategy for 30–39 year old women [70]. A PoC test-time of under one hour from test to result would be ideal, as treatment can follow in the same encounter [22]. In a healthcare worker survey, the participants argued that a sample-to-answer time of less than an hour is indeed optimal [43]. This forms a barrier for most nucleic acid based tests, which take several hours [43]. 

Some researchers therefore argue for more holistic thinking in terms of health services. There needs to be better linkage that connects testing, diagnosis and treatment [66]. Given the large impact LTFU has, resources could also be used in preventing LTFU instead of perfecting diagnostic devices [70].

For example, getting infants on ART could be achieved with combinations of tests of different sensitivity, but proper linkage. The initiation rate of 71% could be achieved with PoC devices with a limited sensitivity of only 72%, but a successful linkage rate of 99%, or with a test of 100% sensitivity and 70% successful linkage [86,88]. Some researchers suggest that PoC tests should be evaluated just as much on their ability to facilitate linkage as they are on their performance [88]. As demonstrated before, the cost-benefit also favors a two-step system. The absence of functional referral systems is seen as a huge roadblock by other authors as well [95].

Therefore, information technology plays a key role in the context of PoC, maybe even more so than new and better devices themselves. The rapid reporting of results and counseling via mobile phones are essential for a decentralized use of PoC. Mobile phone-linked PoC devices can also assist in data capture and quality control, medication distribution, PoC tracking and data storage [5,43,97]. The usage of mobile phones in this way is generally categorized as mHealth, an expanding subfield of eHealth, and concerns itself with the use of wireless technology instead of connection through ordinary landline infrastructure, such as in eHealth. This is especially interesting for LMICs, as mobile phone usage outperforms other communication infrastructure usage [98]. In total, 70% of the 7.4 billion users of cellular phones reside in LMICs, and especially in sub-Saharan Africa, mHealth had a rapid expansion, making this approach hugely promising [20,99,100]. The response to Ebola PoC in the last epidemic might serve as an example, as it was very fast as a result of the effective surveillance systems in place. However, healthcare systems are slow to use this connectivity to their full potential [66]. This is starting to change as mHealth is more utilized. In a review of 255 studies of mHealth applications, 93 studies fell into the realm of health monitoring and surveillance, the second largest group with 88 publications concerned themselves with raising health awareness [98]; another study found the most used context to be increased patient follow-up, as well as patient compliance [100].

### 4.3. Training

While large hospitals in central areas might also have an appropriate workforce, the staff in rural healthcare clinics, which are the main access points to healthcare for the rural population, consist mainly of untrained individuals or inadequately small workforces; often just one doctor, nurse or pharmacist, with the possible addition of lay healthcare workers (LHWs) [43,61,95,101].

Human resources are in surprisingly short supply when it comes to healthcare workers and may stretch out the system, especially in rural sites [26,66]. Additional onsite testing could put even more strain on the already overworked staff; as already mentioned, this might be a major problem if there are no additional staff or incentives available, and might discourage PoC use [20,43,102]. There is also a problem in the lack of educated healthcare personnel, especially in Africa, which has over 24% of the worldwide disease burden while only having 2% of physicians of the world [61,103,104,105]. The shortage of skilled healthcare workers was seen as a problem by several authors [20,49]. 

Surveyed healthcare workers assessed PoC diagnostics as easy to use; however, they still expressed fear of knowledge gaps among their users and concern of incorrect use. For example, the use of a wrong buffer solution or no buffer solution at all was observed [49]. Other researchers also reported reluctance for PoC in LICs due to the need for training, and the costs associated with implementation, as well as diagnosis [45]. It is especially feared that lay healthcare workers will not have the adequate training or knowledge to conduct even simple PoC tests, which could lead to inaccurate results that could damage the perception of PoC in these settings [20]. Despite those fears, the world health organization recommends task shifting to LHWs to meet human resources needs [78,85] and it is a tool that is increasingly used to combat the estimate estimated shortage of 7.2 million healthcare workers, which is the most severe in Sub-Saharan Africa [106].

The question is, can task shifting from healthcare professionals to LHWs be achieved without a loss in reliability of test results. Lay health workers provide an important opportunity to give more people access to healthcare; this is especially the case for rural areas. However, medical devices are usually not designed to account for task shifting [61]. 

Some argue that task-shifting has to be accommodated when designing the PoC device. The interface has to be straightforward and user-friendly, even for laymen, which is often not considered in the design phase [21]. Ideally, the device should be a fully autonomous, robust, “black box” [4], which is fully automated with a simple interface and everything integrated into a simple “sample to answer” process [43]. Others suggest that the technological complexity must be as simple as a home pregnancy test [22], or assess modern PoC diagnostic platforms used for CD4+ testing as too sophisticated for usage in LICs [44]. Laboratory professionals also doubt that diagnostics can be performed by lay-healthcare-workers with appropriate quality assurance [5,45]. This viewpoint might be understandable, given the findings of doctors observing the wrong use of lateral flow devices [49]. The question, therefore, remains whether PoC devices demand usage by healthcare professionals. 

Research is suggesting otherwise. A study about task shifting for the use of the HCTs Pima CD4+ Analyzer (Allere) in Namibia showed that lay-health-workers can produc valid tests as nurses. In a large study of 1429 CD4+ tests, in which 500 were performed by nurses and 929 by LHWs, the reception of test results by the patients was in favor of LHWs, with 98.1% contrary to 95.6%. LHCs were only slightly slower, with a median turnaround time of 21 minutes compared to 20 minutes for nurses. However, both were a tremendous improvement from the turnaround time of a laboratory test, which had a median of 4 days (IRq 2–8). Therefore, task shifting to LHWs may be an appropriate choice, even for more complex tests [85]. Other studies agree that LHWs can perform rapid testing just as well as trained laboratory staff, if trained properly. However, when implementing a training program, it has to be considered that the training package is adapted to the local environment [78]. LHWs in Malawi have named a lack of disease- and job-specific training as a key problem hindering their role as TB care providers [106,107]. Lateral flow tests for HIV testing were so successful in their ease of use, that task shifting could be greatly implemented and the tests in LMIC are now often done by expert-patients or trained lay healthcare workers [95].

#### 4.3.1. Use by Trained Doctors

In cases where trained medical professionals are available, the bottlenecks present themselves differently. One issue is time constraints. In India, doctors prefer clinical diagnoses coupled with empiric treatments over a higher diagnostic security. Broadband antibiotic prescription after only a short symptomatic observation is a common example; this is faster than doing an additional PoC test that might not even be necessary, just in order to make the diagnosis more secure [5]. The general overburden of doctors in India is one factor for this. Generally, visits only last a few minutes, which is generally not enough time for PoC testing. On the doctor’s side, it is better for his reputation to treat several other waiting patients in this time and keep waiting-lines short. Many doctors only have a single room with one nurse as an assistant, even simple lateral flow tests are difficult to conduct under such conditions [5].

Awareness is another issue which is suggested by the literature. Healthcare providers might not be aware of PoC tests on the market [5]. Lack of knowledge about PoC testing among people living in rural areas could be addressed by, for example, advertising and explaining the use of PoC tests for rapid diagnosis of specific diseases. This could raise awareness and prevent last-minute visits to the doctor. However, general awareness of PoC devices in Kenya was surveyed and is high throughout high-, mid- and low-tier healthcare providers and seems to not be a critical barrier. In total, 95% of healthcare providers in his survey could name a disease that can be diagnosed with PoC tests (71% could name two and 24% could name three); only 5% were not able to name any disease which has PoC tests available. However, only 10% of healthcare workers which named more than three PoC devices had actually applied them in their practice [28]. This indicates that the bottleneck is systemic rather than knowledge-based. Higher knowledge and usage about HIV tests were shown by doctors in richer hospitals compared to rural doctors. For example, in malaria diagnostics there is a wide gap between the knowledge about (57%) and actual use of PoC diagnostics (36%), independent of socioeconomic factors. It is suggested that this is because PoC devices for malaria are seen as of limited usefulness as the disease has strong symptoms and the prevalence is foreseeable due to the seasons. Another possible reason might be the lack of availability, the devices not complementing other diagnostic methods or greater success with other diagnostic methods, such as a symptomatic approach. For other identified diagnostics, the knowledge about the device was 1- to 3-times higher than the actual use [28]. Healthcare workers in other studies could identify various PoC tests. The most known ones were for Malaria, HIV, Syphilis, Blood Glucose and Pregnancy. These findings concurred with other surveys [49,108]. 

#### 4.3.2. View on PoC 

With regard to the view of patients on PoC, it was found that, according to healthcare personnel, patients were satisfied with PoC results (97%) and would recommend them (96%). However, only half of clinicians thought they would give reliably accurate results; 46% were unsure and 4% considered them not accurate. In total, 65% of healthcare workers used medication even on a negative test, showing that trust in the test is limited. This concurs with only 20% of healthcare workers stating that they rely on the test alone. The majority sees the test as a complement to other means of diagnosis, such as symptoms [28], as 54% of surveyed encountered barriers preventing PoC use. The likelihood of encountering barriers correlated with hospital tier (45% in high-end hospitals and 53% in mid-tier). In total, 50% of personnel who encountered barriers named reliability issues as a large problem; the second largest obstacle named was availability, with 46%. Only a smaller percentage saw cost (14%) and awareness or training deficiencies (12%) as major obstacles. When asked about improvements to increase PoC use, the respondents replied with improved tests (44%), improved reliability (22%) and standardization (20%) which was specifically mentioned, even though it was not in the survey as an answer. Oddly, increased availability was named by just 22%, despite it being the second highest identified barrier. In total, 85% agreed that PoC is an opportunity for more affordable healthcare in Kenya [28].

**Figure 4:**
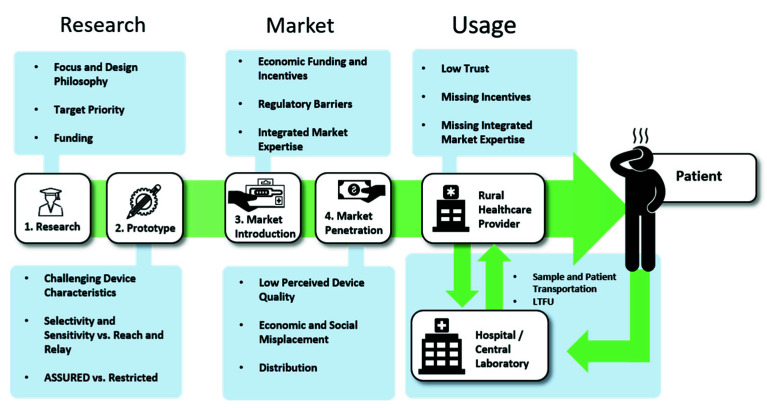
Identified Problems along the value chain.

**Figure 5:**
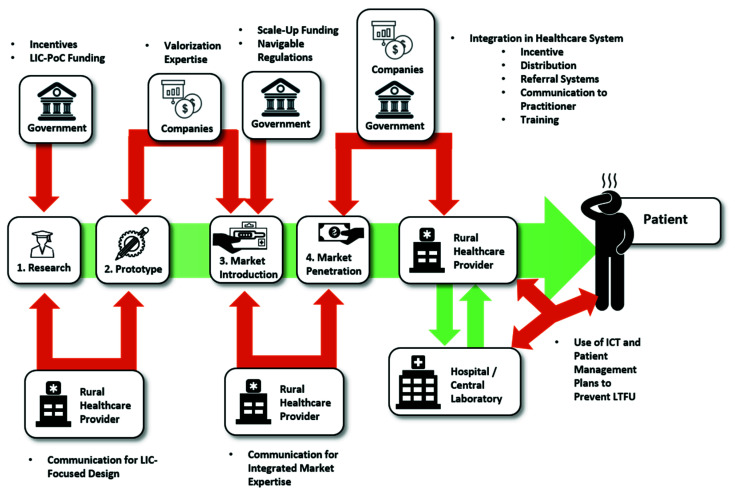
Identified Pivotal Points and Influencing Forces.

From the first start of research in the beginning, to the view of end-users at the patient-side, the bottlenecks of PoC diagnostics along the value chain seem to be as diverse and as different from each other as the actors and circumstances that present themselves on the way, as is summarized in Figure 4. However, as Figure 5 shows, there are many possible solutions at each step as well, which are, next to technological advancements, often based upon the connection of different stakeholders.

## 5. Conclusions

### 5.1. Main Findings

Fundamental research always starts with funding, and it is, therefore, an obvious consideration. However, funding is not only needed here. Additional funding, as well as incentives for valorization is something direly needed to actually make the jump from a research principle to a medical device. Push- and pull-incentives are used with considerable success in drug development and might prove valuable if systems directed at PoC are in place. Connecting all stakeholders, such as research groups, companies, healthcare professionals, as well as governments and NGOs, is essential and enables IP considerations and licenses to be negotiated to everyone’s benefit. Healthcare professionals’ needs can be shared and taken into account, and company expertise in scale-up and distribution can be applied.

For the development of the device, one can ask the question of how important it is to be ASSURED. While many argue for sensitivity, specificity and reliability as a main point, there were other voices arguing for a more integrated view. For example, it can be argued that the needed criteria solely depend on the use-case. This notion has been supported by original research, showing that a two-step system can be cheaper, as well as more specific, despite the first stage not having optimal characteristics. However, for this to work, a proper integration into the healthcare system, with reasonable referral structures and minimal LTFU, has to be achieved. This might be realized by improved ICT structures and the use of mobile phones for diagnostic readouts. Tests can have shortcomings if proper linkage to further tests and treatment are in place and LTFU is at a minimum. However, practitioners need to be aware of the test shortcomings to have a safe basis for decision making. The perceived low device quality can, and does, hinder effective PoC usage due to mistrust by doctors. Clearer communication of what PoC can and cannot achieve might give healthcare providers more certainty in their decision-making and empower them to trust the device and correctly interpret the received results. Clear guidelines for healthcare workers on how to use the results of a PoC device in the grand scheme of things need to be in place, including what the next steps for patients are, either for treatment or further diagnosis. These guidelines need to be beneficial to the healthcare worker as well, instead of just adding additional work. At this moment, it is more in the interest of a doctor in India with 30 waiting patients to simply give the patient antibiotics and send him/her home after a brief symptomatic assessment than to perform a PoC diagnosis, which takes more time. If the patient does not come back, the doctor may assume him cured and treat it as a success. However, this is not in the benefit of society nor the patient. Therefore, test and referral systems must be integrated in a way to lift the burden from the practitioner instead of adding them. 

On the market side, the tremendous influence of a proper placement into the healthcare system and its incentive structure is shown by the use of subpar-PoC devices in India and other countries. If malfunctioning PoC devices can achieve widespread use, surely working diagnostics can be used if they are properly integrated into an incentive structure. The importance of incentive structure is also shown by PoC use in diagnostic laboratories in India and South-Africa, where PoC devices are misguided as cheap laboratory alternatives, instead of their intended goal as a fast, patient-side, diagnostic instrument.

A social problem that was identified as a large bottleneck is the lack of available workforce for testing. While the opinion of researchers regarding task shifting varies from skeptical to enthusiastic, there are interesting insights into its feasibility, arguing that lay-healthcare-workers can even conduct tests on more difficult platforms if trained properly.

### 5.2. Outlook

As research on PoC diagnostics continues, we will get closer and closer to versatile, accurate and cheap detection methods that are more in line with the desirable ASSURED criteria. However, for the immediate success of PoC and for the benefit of patients in LICs, the research part might already be more advanced, compared the other aspects of the value chain. This is shown by the fact that already established PoC tests and resources are not nearly used to their full potential. A wide range of reasons can be identified, from missing funding for scale-up and lack of corporate incentives, as well as delivery problems and stock outs, to the problem of integration in healthcare systems and lacking trust by doctors. The lack of valorization of PoC in LICs seems to be a social and an economical problem, more than a problem of research. 

Researchers will make progress for continuously improved devices, which will be easier to implement. However, the burden of implementation should not be solely put on the shoulders of scientists to discover novel advanced technical solutions for a perfectly ASSURED system. Tests that might not be perfect, but that are instead perfectly adequate for use are in reach, but missing incentive structures and lacking political attention will prevent their effective use. 

While Zipline is a company concerned with infrastructure and not with diagnostics, it still shows, in an impressive manner, how new technology can innovate a whole market, if there is political will and no old stakeholders that benefit from the status quo. In the case of Zipline, there was no real alternative for emergency blood delivery in an appropriate amount of time. The stage was free for Zipline’s new and improved technology. In diagnostics, old infra- and incentive-structures from centralized laboratories to skeptical or time-limited doctors have to be overcome. While developments in research and innovation in PoC diagnostics over recent years are even more impressive than Ziplines drone systems, they also have to conquer larger infrastructural barriers. Zipline’s CEO, Keller Rinaudo, stated that the technology is the easy part. It is more difficult to improve regulatory issues, acquiring and training the necessary workforce locally and creating awareness of their services to doctors and healthcare workers [80]. Mabey et al. provided us with another success story that further demonstrates that PoC tests need to address the whole value chain in order to be successful [109]. They implemented PoC tests for syphilis diagnosis and were successful because they did not only addressed a need in the healthcare system and offered solutions that adhered to the ASSURED criteria, but also managed to impact health care workers training, ensured that effective treatment was available and improved the local medical supply chain. These requirements are further illustrated by the case of blood glucose testing, the most striking example of a PoC success story in HIC. Blood glucose meters and test strips are often unavailable in LICs and even when present, other factors hamper their implementation, such as poor diabetes education and economical constraint regulatory issues [110]. Therefore, sadly, one of the most impactful PoC tools in HICs has not yet been able to achieve the same impact in rural, low-income settings. 

Faced with the healthcare challenges in LICs, a transformation to a smart healthcare system, with real-time information flow and referral structures, will be necessary to make the most out of the innovations that come out of the lab. 

Thus, there is a case to be made that scientific progress and innovation may not be the limiting factor, and other limiting steps seem to be hindering valorization at least as much. Those factors might improve, together with the economic development of LICs, but for now researchers need to take these social factors just as much into account as the technical aspects of new devices. The research and implementation of a new device have to be designed in synergy with its target location, instead of merely adapting to it later on. Advances for fast throughput devices of stable and long lasting reagents with frugal design and the integration of mHealth capabilities, and devices that have task-shifting in mind, will be of great help in this challenge—as long as those technical advancements get connected to the local realities. mHealth connection will only be as potent as the referral structures that are in place to accept it. A task-shifting enabled device is only be as beneficial as the availability of LHWs. Therefore rollout, training and supply plans have to be integrated in and developed together with the device and in close connection to the target environment, which might be, at least in part, an aspect of a researchers work too. Research needs to break down as many barriers and enable the connection of as many stakeholders as possible in order for policy makers and companies to take the leap and bring PoC diagnostics to the patients.
